# PM_2.5_ Exacerbates Oxidative Stress and Inflammatory Response through the Nrf2/NF-κB Signaling Pathway in OVA-Induced Allergic Rhinitis Mouse Model

**DOI:** 10.3390/ijms22158173

**Published:** 2021-07-29

**Authors:** Chun Hua Piao, Yanjing Fan, Thi Van Nguyen, Hee Soon Shin, Hyoung Tae Kim, Chang Ho Song, Ok Hee Chai

**Affiliations:** 1 Department of Pulmonary and Critical Care Medicine, Yantai Yuhuangding Hospital, Yantai 264000, China; chpiao0208@163.com; 2 Department of Anatomy, Jeonbuk National University Medical School, Jeonju 54896, Jeonbuk, Korea; fanyj0915@gmail.com (Y.F.); vandkh1993@gmail.com (T.V.N.); htkim@jbnu.ac.kr (H.T.K.); 3 Division of Food Functionality Research, Korea Food Research Institute, 245 Nongsaengmyeong-ro, Iseo-myeon, Wanju-gun 55365, Jeonbuk, Korea; hsshin@kfri.re.kr; 4 Food Biotechnology Program, Korea University of Science and Technology, Daejon 34113, Korea; 5 Institute for Medical Sciences, Jeonbuk National University, Jeonju 54896, Jeonbuk, Korea

**Keywords:** particulate matter (PM_2.5_), allergic rhinitis (AR), Nrf2, NF-κB, oxidative stress, inflammation

## Abstract

Air pollution-related particulate matter (PM) exposure reportedly enhances allergic airway inflammation. Some studies have shown an association between PM exposure and a risk for allergic rhinitis (AR). However, the effect of PM for AR is not fully understood. An AR mouse model was developed by intranasal administration of 100 μg/mouse PM with a less than or equal to 2.5 μm in aerodynamic diameter (PM_2.5_) solution, and then by intraperitoneal injection of ovalbumin (OVA) with alum and intranasal challenging with 10 mg/mL OVA. The effects of PM_2.5_ on oxidative stress and inflammatory response via the Nrf2/NF-κB signaling pathway in mice with or without AR indicating by histological, serum, and protein analyses were examined. PM_2.5_ administration enhanced allergic inflammatory cell expression in the nasal mucosa through increasing the expression of inflammatory cytokine and reducing the release of Treg cytokine in OVA-induced AR mice, although PM_2.5_ exposure itself induced neither allergic responses nor damage to nasal and lung tissues. Notably, repeated OVA-immunization markedly impaired the nasal mucosa in the septum region. Moreover, AR with PM_2.5_ exposure reinforced this impairment in OVA-induced AR mice. Long-term PM_2.5_ exposure strengthened allergic reactions by inducing the oxidative through malondialdehyde production. The present study also provided evidence, for the first time, that activity of the Nrf2 signaling pathway is inhibited in PM_2.5_ exposed AR mice. Furthermore, PM_2.5_ exposure increased the histopathological changes of nasal and lung tissues and related the inflammatory cytokine, and clearly enhanced PM_2.5_ phagocytosis by alveolar macrophages via activating the NF-κB signaling pathway. These obtained results suggest that AR patients may experience exacerbation of allergic responses in areas with prolonged PM_2.5_ exposure.

## 1. Introduction

Atmospheric particulate matter (PM) is a complex mixture of inorganic substances (such as oxides of transition metals), dust, smoke, metal elements, various liquid and solid substances, as well as biological components including bacteria, fungi, and viruses [1]. Acute and chronic exposure to PM, especially the fine particles (aerodynamic diameters less than or equal to 2.5 μm, PM_2.5_), are harmful to human health because they can penetrate into the bronchi and alveoli of the respiratory tract and cause lung and cardiovascular diseases [2,3,4].

Recently, increasing epidemiologic studies have demonstrated that PM exposure is closely associated with morbidity and mortality due to chronic respiratory diseases [5]. Practical evidence gathered through environmental and epidemiological studies shows a strong association between fine particulate air pollution and health problems such as respiratory illnesses including respiratory tract inflammation, asthma, acute bronchitis, and lung cancer [6,7].

The prevalence of allergic diseases, including asthma, allergic rhinitis (AR), and food allergies, has risen sharply in recent years [8]. AR is an immunoglobulin E (IgE)-mediated, nasal mucosal hypersensitivity reaction to allergens such as pollen, molds, animal dander, and dust mites [9]. Symptoms include sneezing, rhinorrhea, and nasal congestion and irritation [9]. There is increasing evidence from epidemiological and laboratory-based studies that exposure to air pollutants can play an important role in the nasal symptoms of allergic and non-allergic airway diseases.

The transcription factors involved in the oxidative stress response are nuclear factor erythroid 2–related factor 2 (Nrf2) and nuclear factor κB (NF-κB) [10]. The Nrf2/HO-1 signaling pathway is known to play an important role in the regulation of oxidative stress [11]. The transcription factor NF-κB plays an important role in a wide range of inflammatory diseases [12]. The interaction between Nrf2 and NF-κB is interesting because numerous phytochemicals that have anti-inflammatory, anti-oxidative, or anti-cancer properties suppress NF-κB signaling and activate the Nrf2 pathway [13]. Malondialdehyde (MDA) is known to be a feature of oxidative stress [14]. The MDA and Nrf2/HO-1 axis are well known as the key elements of oxidative stress and antioxidant balance [15]. Nrf2 is a transcription factor that regulates the expression of multiple antioxidant and cytoprotective proteins and enzymes, thus playing a key role in protection against oxidative damage [15]

Although many toxicological studies have been conducted to clarify the biological response to PM_2.5_, there is no evidence to elucidate the clear molecular mechanism of PM_2.5_ mediated toxicity through the Nrf2/NF-κB signaling pathway. Therefore, the purpose of this study was to evaluate the direct impact of long-term PM_2.5_ exposure on the oxidative stress and inflammatory response through the Nrf2/NF-κB signaling pathway in an ovalbumin (OVA)-induced AR mouse model. The involvement of ROS-dependent MDA production and SOD activity were also evaluated. PM_2.5_-induced oxidative stress has been considered as a key molecular mechanism of PM_2.5_-mediated toxicity [16].

The present study demonstrated that the PM_2.5_/OVA group is strongly toxic to AR and can induce oxidative through MDA production. Evidence was also provided, for the first time, that the activity of Nrf2 signaling pathway decreased in PM_2.5_ exposed AR mice. Furthermore, the PM_2.5_ exposed group increased pathological changes, related to the inflammatory cytokine, and clearly enhanced PM_2.5_ phagocytosis by alveolar macrophages.

## 2. Results

### 2.1. Effect of PM_2.5_ on the Allergic Symptoms in OVA-Induced AR Mouse

**Figure 1:**
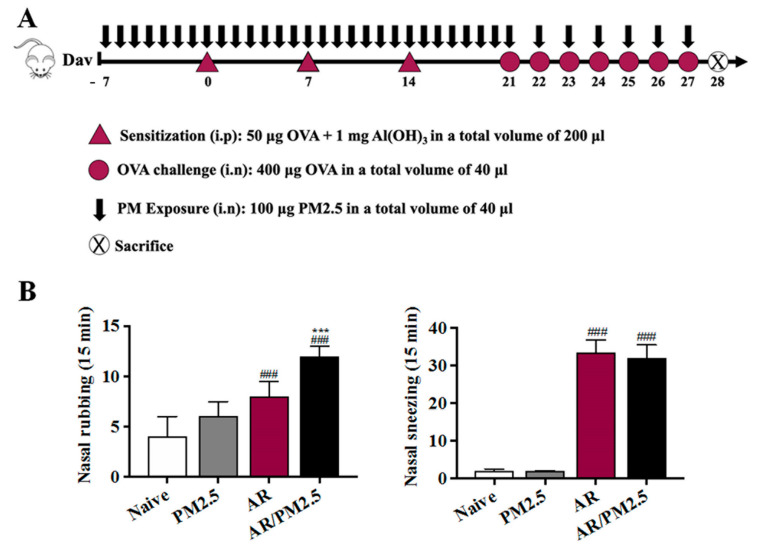
Protocol for the establishment of the AR mouse model and PM_2.5_ exposure and effect of PM_2.5_ on the allergic symptoms in OVA-induced AR mice. (**A**) Protocol for establishment of the AR mouse model. (**B**) Nasal rubbing and sneezing events were counted for 15 min after the last OVA challenge. The values represent the mean ± SEMs. Significant differences at *### p* < 0.001 compared with the Naive group. *** p < 0.001 compared with the AR group.

To determine the role of PM_2.5_ exposure in AR, the frequency of allergic symptoms such as nasal rubbing and sneezing per groups was measured. The incidence of nasal rubbing and sneezing of mice in the AR group increased significantly compared with the Naive group, and more severe symptoms occurred in the AR/PM_2.5_ group (Figure 1B). Notably, the data showed that mice treated with PM_2.5_ displayed a marked up-regulation effect of allergic symptoms (Figure 1B).

### 2.2. Effect of PM_2.5_ on the Infiltration of Total and Inflammatory Cells in NALF and BALF of OVA-Induced AR Mouse

**Figure 2:**
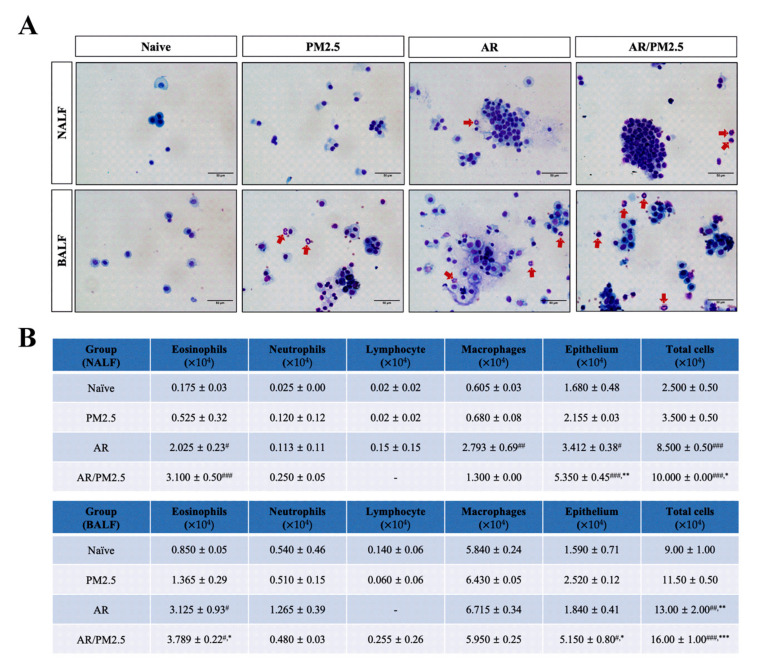
Effect of PM_2.5_ on the infiltration of total and inflammatory cells in NALF and BALF of OVA-induced AR mice. (**A**) The differential cells were isolated using cytospin and then stained with Diff-Quik. Red arrows indicate eosinophils. (**B**) Total and differential cell numbers were counted using a hemocytometer. The values represent the mean ± SEMs. Significant differences at *# p* < 0.05, *## p* < 0.01, *### p* < 0.001 compared with the Naive group. * p < 0.05, ** p < 0.01, *** p < 0.001 compared with the AR group.

To explore the role of PM on allergic inflammation, the inflammatory cell levels in cytospin preparations of NALF and BALF were measured followed by Diff-Quik staining. The results showed that inflammatory cells increased in OVA-induced AR mice compared with those in the Naive group, especially eosinophils, and these reactions were exacerbated by AR with exposure to PM_2.5_ (Figure 2A,B).

### 2.3. Effect of PM_2.5_ on the Allergic Responses with OVA-Specific Antibodies Level in Serum of OVA-Induced AR Mouse

**Figure 3:**
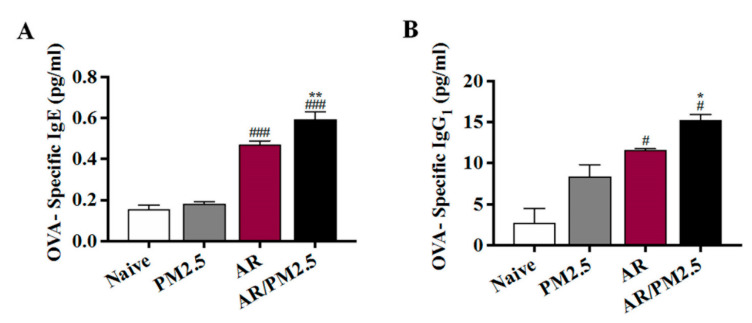
Effect of PM_2.5_ on the allergic responses with OVA-specific antibodies level in serum of OVA-induced AR mouse. (**A**) The levels of OVA-specific IgE and (**B**) OVA-specific IgG_1_ in serum. The values represent the mean ± SEMs. Significant differences at *# p* < 0.05, *### p* < 0.001 compared with the Naive group. * p < 0.05, ** p < 0.01 compared with the AR group.

To demonstrate whether an allergic response was detectable systemically, OVA-specific IgE and OVA-specific IgG_1_ levels in serum were measured. Mice in the AR and AR/PM_2.5_ groups displayed increased levels of OVA-specific IgE and OVA-specific IgG_1_ relative to mice in the Naive group. Moreover, these levels were upregulated by the PM_2.5_-exposed AR group (Figure 3A,B).

### 2.4. Effect of PM_2.5_ on the Alveolar Macrophage function in BALF and Lung Tissue of OVA-Induced AR Mouse

**Figure 4:**
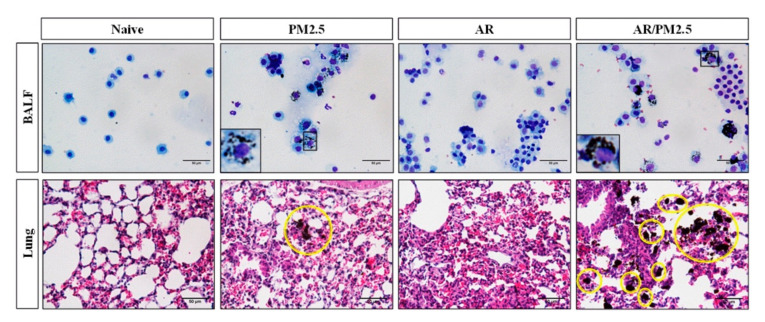
Effect of PM_2.5_ on the alveolar macrophage function in BALF and lung tissue of OVA-induced AR mice. Airway macrophages were visualized by light microscopy. The area occupied by black material (PM_2.5_) in each macrophage was evaluated in BALF and lung tissue with H&E staining. Yellow circles indicate the PM_2.5_ phagocytosis. Scale bars: 50 μm.

When the lungs are exposed to particulates, the particulates are engulfed by phagocytes, such as alveolar macrophages, to be removed from the deep of the lungs (12). Therefore, we first investigated the effects of particulates on the alveolar macrophage function. There was no significant difference in the pattern of PM_2.5_ loading in airway macrophages with the AR and Naive groups. Interestingly, PM_2.5_ exposure, such as in the PM_2.5_ and AR/PM_2.5_ groups, detected PM phagocytosis by alveolar macrophages in BALF and the lung tissue, and these effects were markedly aggravated by AR with PM_2.5_ exposure (AR/PM_2.5_) group (Figure 4).

### 2.5. Effect of PM_2.5_ on the Oxidative Stress and Nrf2/NF-κB Signaling Pathway in OVA-Induced AR Mouse

**Figure 5:**
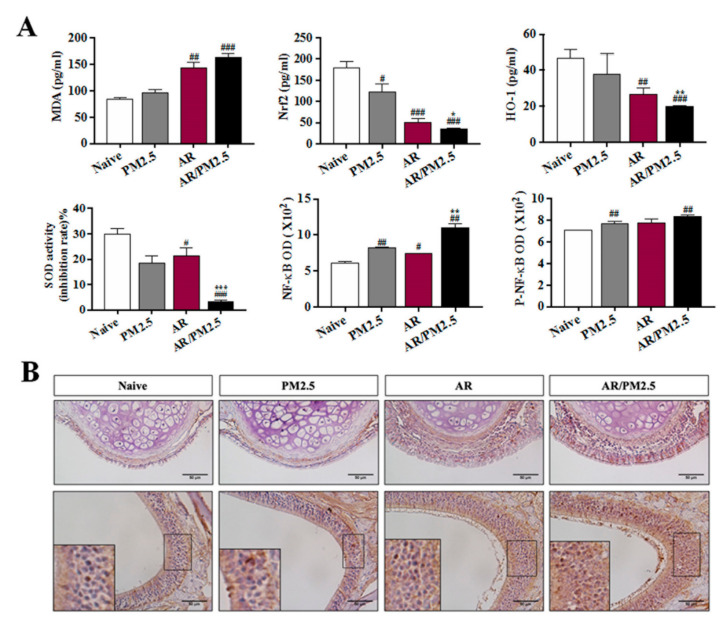
Effect of PM_2.5_ on the oxidative stress and the Nrf2/NF-κB signaling pathway in OVA-induced AR mice. (**A**) The levels of MDA production, Nrf2 active forms, antioxidant enzyme SOD and HO-1 productions, and NF-κB and P-NF-κB in NALF. (**B**) Immunohistochemical assay of NF-κB. The values represent the mean ± SEMs. Significant differences at *# p* < 0.05, *## p* < 0.01, *### p* < 0.001 compared with the Naive group. * p < 0.05, ** p < 0.01, *** p < 0.001 compared with the AR group.

The MDA and Nrf2/HO-1 axis are the key elements of oxidative stress and antioxidant balance [14,15]. In order to investigate the potential oxidative toxicity mechanism of PM_2.5_ in OVA-induced AR, we monitored and tested the temporal accumulation of MDA and SOD production through the Nrf2/HO-1 pathway that was associated with antioxidant effects. The results revealed that the level of MDA significantly increased by AR with PM_2.5_ exposed group, followed by down-regulation of SOD activity and production of HO-1 in NALF, which subsequently induced the oxidative stress in AR (Figure 5A). These findings suggested that the PM_2.5_-induced toxicological effect may contribute to increased oxidative stress and inflammatory response in AR.

Next, to quantify the aggravated effects of NF-κB observed in NALF using an ELISA kit, NF-κB nuclear translocations were performed by immunohistochemical staining. Immunohistochemical staining readily provided a quantitative measure of NF-κB expression in nasal mucosa. NF-κB in each group was found to be mainly expressed in the nucleus and stained with a brown color. The results revealed the predominance of NF-κB-stained cells in nasal epithelium and mucosa from mice in our OVA-induced AR compared with the Naive group (Figure 5B). In addition, the AR with PM_2.5_ exposure (AR/PM_2.5_) group increased the prevalence of more significant stained cells in nasal tissue (Figure 5B). These results suggested that PM_2.5_ exposure activated the NF-κB and subsequent translocation from the cytoplasm into the nucleus to further exacerbate the allergic inflammation induced by OVA.

### 2.6. Effect of PM_2.5_ on the Histological Changes in the Nasal and Lung Tissues of OVA-Induced AR Mouse

**Figure 6:**
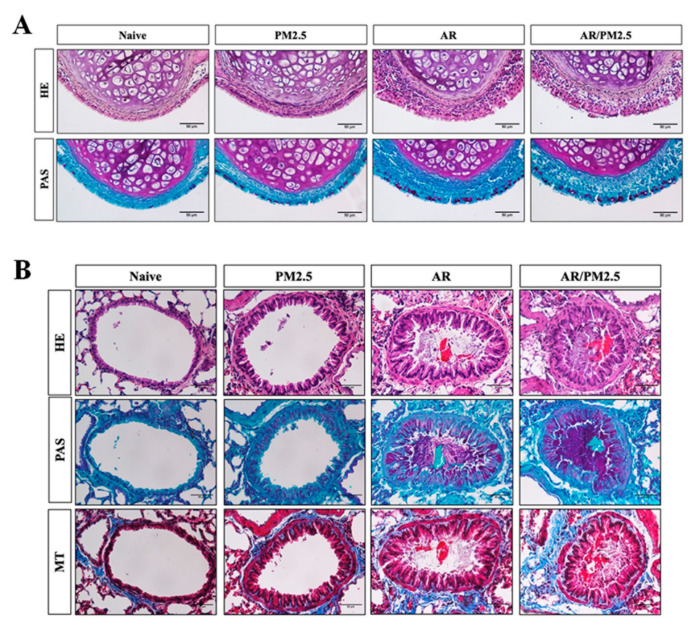
Effect of PM_2.5_ on the histological changes in the nasal and lung tissues of OVA-induced AR mice. (**A**) Histological examinations in nasal tissue by H&E and PAS staining. (**B**) Histological examinations in lung tissue by H&E, PAS, and Masson trichrome staining. Scale bars: 50 μm.

The nasal epithelium served as the first line of defense of the upper airways against a variety of inhaled particles that produce harmful effect the on the hosts, including PM_2.5_ [17]. In the present study, we examined the altered allergic airway inflammation using histology analysis of inflammation and goblet cell hyperplasia in the nasal mucosa following the HE and PAS staining. Compared with the Naive group, OVA-induced AR group increased inflammatory infiltration and epithelium disruption and goblet cell hyperplasia. Additionally, AR with the PM_2.5_ exposure group obviously aggravated these effects (Figure 6A).

Inflammatory cell infiltrations, goblet cell hyperplasia, and collagen deposition in lung tissue were evaluated using H&E, PAS, and Masson trichrome staining, respectively. Representative images of lung tissues are shown in Figure 6B; OVA-induced AR exhibited a significant increase the inflammatory infiltration and epithelium thickening, goblet cell hyperplasia and mucus overproduction, accompanied by the collagen deposition and tissue fibrosis compared with the Naive group. After exposure to PM_2.5_, a substantial aggravation of these effects was observed compared with the AR. The histological changes were minimal in the group exposed to PM_2.5_ alone.

### 2.7. Effect of PM_2.5_ on the Differentiation Helper T cells Responses in NALF of OVA-Induced AR Mouse

**Figure 7:**
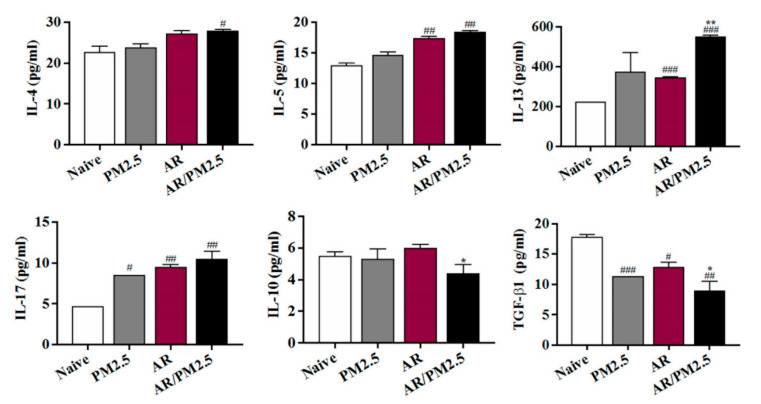
Effect of PM_2.5_ on the differentiation helper T cells responses in NALF of OVA-induced AR mice. The levels of the Th2, Th17, and Treg cell effector cytokines including IL-4, IL-5, IL-13, IL-17, IL-10, and TGF-β1 in NALF were measured. The values represent the mean ± SEMs. Significant differences at # *p* < 0.05, ## *p* < 0.01, ### *p* < 0.001 compared with the Naive group. * *p <* 0.05, ** *p* < 0.01 compared with the AR group.

Levels of the Th2, Th17, and Treg cell effector cytokines IL-4, IL-5, IL-13, IL-17, IL-10, and TGF-β1 in NALF were measured. As shown in Figure 7, compared to the untreated AR group, Th2 cytokines including IL-4, IL-5, and IL-13 productions in AR animals treated with PM_2.5_ were significantly increased (Figure 7). Recently, the discovery of Th17 and Treg cells added a layer of complexity to the older Th1/Th2 balance paradigm and understanding of the pathogenesis of AR [18]. However, the exact contributions of Th17 and Treg cells to the nasal mucosal status of OVA-induced AR mouse model with PM_2.5_ exposure have not been thoroughly investigated and thus remain controversial. The prevalence and function of Th17 cells were significantly upregulated, and Treg cells were downregulated. Our results showed that the IL-17 level in AR animals treated with the PM_2.5_ was significantly increased compared with the AR group. Moreover, the PM_2.5_ exposed group increased the IL-10 and TGF-β1 levels as compared with the AR group (Figure 7).

## 3. Discussion

AR is a chronic inflammatory disease of the nasal mucosa caused by inheritable and environmental factors [19]. PM_2.5_ enters the human body more easily than inhalable particulate matter, and the toxic substances carried are more toxic. The higher the PM_2.5_ concentration in the environment, the more serious damage to human health [20]. Many studies have been conducted on the correlation between PM and health effects, and PM has shown to have a negative effect on human respiratory diseases due to its small size; PM_2.5_ can penetrate deep into the alveoli of the lungs and potentially into the blood circulation.

There is strong evidence that PM_2.5_ can exacerbate AR and increase the number of daily medical consultations related to AR [21]. The aim of this study was to investigate the effect of PM_2.5_ on allergic airway inflammation and its molecular mechanism. In the present study, it was observed that PM_2.5_ enhanced OVA-induced allergic symptoms such as nasal rubbing and sneezing. Moreover, there was an increase in the total cells as well as epithelium, eosinophils, and macrophages in the PM_2.5_-exposed AR group in NALF (Figure 3). Alveolar macrophages are the first line of defense against foreign substances entering the lower airways and are essential for clearing dust from lung [22]. This study suggested that the PM_2.5_-exposed group exhibited PM_2.5_ phagocytosis by alveolar macrophages, and it was more severe in AR than with the PM_2.5_-exposed group (Figure 4). These finding suggests that the phagocytosis of PM_2.5_ by airway macrophages may be aggravated in OVA-induced AR.

Oxidative stress is caused by the overproduction and accumulation of ROS that overwhelms the already impaired antioxidant defense mechanism in the form of cellular injury [23]. Some studies show that low doses of PM_2.5_ can cause significant oxidative stress and inflammation in mice [24]. The biological activation of PM_2.5_ was used to evaluate the oxidative stress. It was found that PM_2.5_ aggravated the oxidative stress by increasing the MDA production, further inhibiting the Nrf2 signaling activation and decreasing the antioxidant enzyme HO-1 and SOD (Figure 5A). These results showed that exposure to PM_2.5_ reduced the antioxidant capacity, increased the lipid peroxidation, and induced the oxidative stress relative to the OVA-induced AR.

NF-κB signaling plays an important role in regulation of inflammatory responses [25]. Oxidant/antioxidant imbalance in the lung leads to the activation of the NF-κB [26]. Some studies have suggested that NF-κB plays an essential role in PM-induced pathogenesis of diseases [27]. It was investigated whether NF-κB affected in PM exposed AR. The data showed that the NF-κB signaling was activated by the PM_2.5_ exposure (Figure 5A). In addition, NF-κB activation by nuclear translocation plays a central role in inflammation. Immunohistochemical analysis showed that the PM_2.5_ exposure markedly increased the NF-κB stained cell accumulation in nasal tissue (Figure 5B). Here, the present results demonstrated that NF-κB is activated and NF-κB translocated from the cytoplasm to the nucleus after PM_2.5_ exposure.

In addition, the imbalance of CD4+ T-cell subsets, which include Th2, Th17, and Treg cells, has been implicated in the etiology of PM_2.5_-induced airway inflammation [28]. Treg cells generally release TGF-β and IL-10 to suppress other Th cells, and the function of Treg may be impaired in allergic disease [29,30]. The imbalance of Th17/Treg is associated with the development of inflammatory and autoimmune diseases such as AR and asthma [31,32]. IL-17 expression in the nasal mucosa was associated with nasal eosinophilia and the clinical severity of AR [33]. The results of the present study showed that the OVA-induced AR group with PM_2.5_ exposure increased the Th2 and Th17 cells as well as IL-4, IL-5, IL-13, and IL-17, but decreased Treg cells as well as TGF-β1 expression (Figure 7). Thus, the findings of the present study suggested that PM_2.5_ exposure has a greater toxic effect on OVA-induced AR mice.

The airway epithelium plays a critical role in forming a physical barrier, which protects submucosal tissue from inhaled harmful substances [34]. A recent study indicated that airborne PM may directly cause airway epithelial cell injury [35]. To further characterize the induced airway inflammation, we accessed the airway inflammation with nasal and lung histopathology. The most important nasal inflammation observed in this study included the epithelium disruption, inflammatory cell infiltration, and mucus production, resulting in an increase of goblet cell hyperplasia of AR (Figure 6A). After PM_2.5_ exposure, these effects were aggravated. In addition, collagen is a main structural framework of tissue, and collagen deposition is essential for tissue remodeling after damage or inflammation [36]. In the present study, the degree of collagen deposition and goblet cell hyperplasia were significantly enhanced in lung tissue in OVA-induced AR exposed to PM_2.5_ compared with AR, as well as nasal tissue inflammation (Figure 6B).

## 4. Materials and Methods

### 4.1. Animals

Six-week-old male BALB/c mice were purchased from Damool Science (Dae-jeon, Korea). Mice were housed in a laminar air-conditioned room maintained at 23 ± 2 °C at a relative humidity of 55 ± 10% with a 12 h dark/light cycle. All experiments were controlled in accordance with the care and use of experimental animals and were approved by the Institutional Animal Care and Use Committee of Jeonbuk National University Laboratory Animal Center (CBNU 2019-071, 2 October 2019).

### 4.2. PM_2.5_ Sample Preparation and Chemical Analysis

PM_2.5_ (Diesel particulate matter NIST standard reference material 2975, Darmstadt, Germany) was purchased from Merck, and concentrated and diluted with sterile saline. The PM_2.5_ was stored at 4 °C. The main polycyclic aromatic hydrocarbons (PAHs) and nitro-substituted PAHs in diesel particulate matter (NIST, 2009) are shown in Table 1.

### 4.3. Mouse Model of AR and PM_2.5_

An OVA-induced AR mouse model was established according to the method described by Kim et al. with some modifications [10]. In brief, OVA (grade VI; Sigma, St. Louis, MI, United States) was used to sensitization and challenge the mice. Thirty BALB/c mice were randomly divided into the following four groups: Naive, AR, PM_2.5_, and AR/PM_2.5_. PM_2.5_ samples were suspended in saline at 2.5 mg/mL (100 μg/animal/time). Mice were immunized with an intraperitoneal injection of 50 μg OVA with 1 mg aluminum hydroxide gel (Imject Alum; Thermo Scientific, Rockford, MD, USA) on days 0, 7, and 14. Thereafter, prolonged continuous inflammation was maintained followed by a daily intranasal challenge with OVA diluted in sterile normal saline (400 μg OVA/20 μL per mouse) from days 21 to 27 (Figure 1). Those in the Naive group and saline/PM groups were treated with an equal volume of saline instead of OVA using the same protocol. The mice in both PM_2.5_ groups were treated via an anterior nasal cavity drop (20 μL/nostril) once a day for a successive period of 35 days during days 7 to 27 (Figure 1A). The mice were sacrificed under general anesthesia on day 28 following the last OVA intranasal challenge.

### 4.4. Allergic Symptom Scores

On day 27, after the final intranasal OVA challenge, nasal symptoms were recorded for 15 min to count the frequency of nasal rubbing and nasal sneezing and experimental conditions were counted by blinded observers.

### 4.5. Collection and Analysis of Nasal Lavage Fluid (NALF) and Bronchoalveolar Lavage Fluid (BALF)

After 24 h of the last OVA challenge, the trachea was opened and incised, and then 1 mL saline was flushed into the nasal cavity for collection of NALF. Lavage fluids were centrifuged at 1000× *g* for 10 min at 4 °C, and the supernatant was separated and stored at −80 °C for further analysis. Lavage cells were resuspended in 150 μL saline and then counted with a Hemocytometer. For classification and counting of cell, smear preparations were made and stained by Diff-Quik (Sysmex Co., Kobe, Japan). BALF was collected two times by flushing 1 mL of saline into the lung via a tracheal cannula. Lavage fluids were centrifuged at 1000× *g* for 10 min at 4 °C and the lavage supernatant was separated and stored for further analysis. Inflammatory cells were differentiated into macrophages, eosinophils, lymphocytes, and neutrophils according to standard morphology, and counted over 100 cells at ×200 magnification with a light microscope (Leica, München, Germany).

### 4.6. PM_2.5_ Content of Airway Macrophage

Airway macrophages were visualized by light microscopy. The area occupied by black material (PM_2.5_) in each macrophage was evaluated in BALF and lung tissue with H&E staining as previously described above [37].

### 4.7. Collection and Measurement of Serum

Briefly, under deep ether anesthesia, the blood samples were collected via the retroorbital plexus. Serum samples were separated by centrifugation at 1000× *g* for 10 min at 4 °C, and then stored at −80 °C until used. The concentration of serum OVA-specific IgE and OVA-specific IgG_1_ was determined by using an ELISA kit, and the optical density (OD) was measured at 450 nm in accordance with the protocol provided by the manufacturer.

### 4.8. Tissue Preparation

Nasal samples from the incisors to the mucosa of nasal septum were harvested on day 29 for histological analysis. The nasal samples and lung lobes were gently removed and, after 3 days, fixed in 10% (*v*/*v*) paraformaldehyde, then the nasal samples were decalcified for 2 days in an ethylenediamine triacetic acid, and the specimens were embedded in paraffin wax.

### 4.9. Histological Examinations

Histological examination of inflammatory changes in nasal mucosa and lung tissues was performed in each animal. Lung samples were sliced into 4 μm sections. The nasal samples were cut at the level of the incisive papilla of the hard palate in coronal sections for examinations of the nasal mucosa. Four-micrometer thick paraffin sections were deparaffinized in xylene and rehydrated in ethanol before hematoxylin and eosin (H&E) staining for evaluation of the airway inflammation, such as the nasal mucosa and lung tissue. Periodic acid-Schiff (PAS) staining was employed for visualizing the development of goblet cell hyperplasia. Granules in the cytoplasm and a two-lobed nucleus were counted under a microscope. Masson trichrome staining was used to reveal the sub-epithelial deposition of collagen in the lung tissue. Positive trichrome-stained areas were used to assess the degree of the sub-epithelial fibrosis. The nasal mucosa in the posterior part of nasal septum and lung tissue was determined under a light microscope in a blinded manner and observed at a high-power filed (×400).

### 4.10. Immunohistochemistry

For the immunohistochemistry assay, paraffin sections were deparaffinized, dehydrated, and washed in PBS (10X) with 0.3% Tween-20 buffer (PBST), and aprotein block was applied and incubated for 10 min at room temperature to nonspecific background staining. Slides were microwaved with a citrate anhydrous buffer (pH 6.0) to facilitate antigen retrieval. Subsequently, slides were incubated with a primary mouse anti-rabbit NF-κB (Cell Signaling Technology, Danvers, MA, USA) antibody (1:400 dilution) overnight at 4 °C. After removal of primary antibodies, sections were washed and incubated with biotinylated secondary antibody at room temperature for an hour and a half, followed by an avidin-biotin-peroxidase complex (Vector Laboratories, Burlingame, CA, USA). After the excess secondary antibody was removed, sections were washed with PBST and incubated with DAB solution for 1 min (1:50; Millipore, Billerica, MA, USA). Sections were rinsed in distilled water and dehydrated to terminate the reaction, and the cover was slipped for microscopic examination.

### 4.11. Enzyme-Linked Immunosorbent Assay (ELISA)

The concentrations of Interleukin (IL)-4, IL-5, IL-10, IL-13, IL-17 and TGF-β1 in NALF were measured. In addition, supernatant was obtained for analysis of NF-кB, phosphorylation (p)-NF-кB, and the levels of Nrf2 and HO-1 in NALF were measured by ELISA in accordance with the manufacturer’s instructions (R&D Systems, Minneapolis, MN, USA). Supernatants were tested for lipid peroxidation by collecting and measuring the concentration of MDA. The activity of Superoxide dismutase (SOD) was analyzed and the OD value was read at a wavelength of 450 nm on a microplate reader. All assays were performed in triplicate. The concentration of each protein was calculated from the standard curve.

### 4.12. Statistical Analysis

Results were analyzed using Graph Pad Prism software (v5.0, La Jolla, CA, USA) and expressed as mean ± SEMs over the number of experiments. Statistical analysis was adjusted to Student’s *t*-test and ANOVA with Dunnett’s test. Statistical significance was defined at the 95% confidence level (*p* < 0.05).

## 5. Conclusions

In summary, it is very meaningful to clarify the toxicity mechanisms caused by the co-exposure of PM_2.5_ and OVA-induced AR. The present study suggested that PM_2.5_ presented a toxic effect to OVA-induced AR and induced oxidative stress by the Nrf2/HO-1-mediated signaling pathway. In addition, these results provided evidence that MDA production increased, and decreased the antioxidant enzymes such as SOD and HO-1 after PM_2.5_ exposure. Interestingly, PM_2.5_ can activate the NF-κB pathway and induce the inflammatory responses by increasing the inflammatory mediators.

Taken together, these results indicated that PM_2.5_ could aggravate AR by targeting Nrf2/HO-1 and the NF-κB signaling pathway. These findings suggest that PM_2.5_-induced toxicological effects may contribute to increased oxidative stress and inflammatory response in AR. All these findings will help develop new strategies for the prevention and treatment of diseases associated with PM_2.5_ exposure.

## Figures and Tables

**Table 1:** Mean concentration (mg/kg) of polycyclic aromatic hydrocarbons (PAHs) and nitro-substituted PAHs (nitro-PAHs) detected in the PM_2.5_ samples.

PAHs	Mass Fraction (mg/kg)	Nitro-PAHs	Mass Fraction (mg/kg)
2-Methylphenanthrene	2.22 ± 0.21	9-Nitrophenanthrene	0.466 ± 0.013
1-Methylphenanthrene	0.923 ± 0.057	3-Nitrophenanthrene	0.190 ± 0.007
Triphenylene	5.32 ± 0.24	2-Nitrofluoranthene	0.231 ± 0.032
Benzo[j]fluoranthene	0.819 ± 0.093	3-Nitrofluoranthene	3.80 ± 0.24
Benzo[a]fluoranthene	0.066 ± 0.007	1-Nitropyrene	35.2 ± 2.2
Dibenz[a]anthracene	0.523 ± 0.047	7-Nitrobenz[a]anthracene	3.57 ± 0.32
Picene	0.902 ± 0.091	6-Notrochrysene	2.45 ±0.33
Dibenzo[a,e]pyrene	0.599 ± 0.024		
Dibenzo[b,k]fluoranthene	2.54 ± 0.08		

## Data Availability

The data used to support the findings of this study are available from the corresponding author upon request.
